# A parasitic leiomyoma of the sigmoid mesentery with schwannoma-like image findings

**DOI:** 10.1186/s40792-024-02015-4

**Published:** 2024-09-10

**Authors:** Koki Fujiwara, Chisato Takagi, Michio Sato, Toshiki Tokuda, Masato Tomita, Atsunori Sugita, Kohei Furuya, Makoto Jinushi, Toshiyuki Mitsuya, Nobutoshi Ando

**Affiliations:** 1 Department of Surgery, International Goodwill Hospital, 1-28-1, Nishigaoka, Izumi-Ku, Yokohama, Kanagawa 245-0006 Japan; 2 Department of Radiology, Keio University Hospital, 35, Shinanomachi, Shinjuku-Ku, Tokyo, 160-0016 Japan; 3 Department of Obstetrics and Gynecology, International Goodwill Hospital, 1-28-1, Nishigaoka, Izumi-Ku, Yokohama, Kanagawa 245-0006 Japan; 4 Department of Pathology, International Goodwill Hospital, 1-28-1, Nishigaoka, Izumi-Ku, Yokohama, Kanagawa 245-0006 Japan

**Keywords:** Parasitic leiomyoma, Laparoscopic myomectomy, Schwannoma

## Abstract

**Background:**

Parasitic leiomyoma (PL) consists of uterine fibroids separate from the uterus that grow in extrauterine tissues such as the peritoneum and mesenterium. The diagnosis of PL requires a thorough medical history of laparoscopic myomectomies using a morcellator and the identification of typical magnetic resonance imaging (MRI) findings as uterine fibroids. Imaging diagnosis of PL is occasionally difficult when PL degenerates in various ways, owing to atypical findings on computed tomography (CT) and MRI.

**Case presentation:**

A 29-year-old woman with a history of laparoscopic myomectomy visited a local hospital with lower abdominal pain. A mesenteric tumor on the sigmoid mesentery was suspected on MRI, and she was referred to our hospital. CT scan showed strong early contrast uptake in the center of the tumor, and MRI T2-weighted images showed high signals at the tumor margins and low signals in the center, suggesting a schwannoma. PL was also part of the differential diagnosis because of the patient’s history of laparoscopic myomectomy. With a preoperative diagnosis of a sigmoid colon mesenteric tumor undeniably of malignant origin, laparoscopic resection of the sigmoid mesenteric tumor was performed. Histopathological examination revealed it to be a PL.

**Conclusions:**

We report a case of PL of the sigmoid mesentery with schwannoma-like findings on imaging that was treated laparoscopically. PL is sometimes difficult to distinguish from schwannomas because of the variety of imaging findings, such as uterine fibroids. PL should be considered in the differential diagnosis of mesenteric tumors following laparoscopic myomectomies, even if it does not show typical imaging findings, such as uterine fibroids.

## Background

Parasitic leiomyomas (PL) comprise uterine fibroids detached from the uterus that proliferate in extrauterine tissues, such as the peritoneum and mesentery, leading to diverse symptoms, including pelvic pain [1]. Iatrogenic PLs have recently been reported after laparoscopic myomectomy with morcellation [2, 3]. The diagnosis of PL requires a thorough medical history of laparoscopic myomectomy using a morcellator and the identification of typical magnetic resonance imaging (MRI) findings as uterine fibroids. PL is difficult to diagnose when tissue degeneration results in atypical imaging findings. Herein, we present a case of PL originating in the sigmoid mesentery characterized by atypical imaging findings during the preoperative diagnostic process.

## Case presentation

A 29-year-old woman presented to a local hospital with lower abdominal pain. A mesenteric tumor was suspected on MRI and she was referred to our hospital. She underwent a laparoscopic myomectomy for a uterine myoma at another hospital at 25 years of age. According to the operational records of the former hospital, surgical scissors were used instead of morcellators to shred the leiomyoma in the collection bag. The specimen measured 100 mm × 90 mm and weighed 177 g. Four laparoscopic myomectomy surgical scars were observed at the umbilicus, left lower abdomen, center of the umbilical pubis, and right lower abdomen. The mass was not palpable.

### Preoperative examination

The serum levels of carbohydrate antigen 19-9 (CA 19-9), carcinoembryonic antigen (CEA), CA-125, and soluble interleukin-2 receptor (sIL-2R) were ≤ 1.2 U/mL, 0.6 ng/mL, 9.5 U/mL, and 206 U/mL, respectively, which were all within normal range. Other laboratory test results were also within normal ranges.

**Fig. 1:**
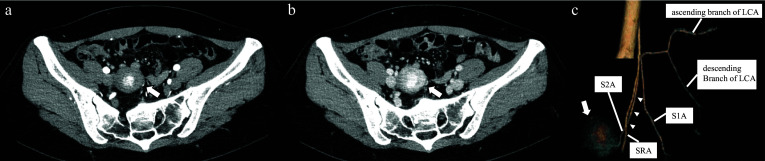
Contrast-enhanced computed tomography (CT) of the abdomen and the pelvis. A 29-year-old woman has a well-defined tumor, 3.5 cm in size, on abdomino-pelvic imaging contrast-enhanced CT. **a** Central contrast-enhancement in the early phase and **b** contrast-enhanced margins in the late phase. **c** 3D-CT angiography showing the mesenteric tumor (arrow) apparently fed by a branch of the S2A (arrow head). *LCA* left colic artery, *S1A* first sigmoidal artery, *S2A* second sigmoidal artery, *IMA* inferior mesenteric artery, *SRA* superior rectal artery

Contrast-enhanced computed tomography (CT) revealed a well-defined tumor measuring 38 mm × 32 mm × 42 mm in diameter in the pelvic cavity. The tumor was centrally contrast-enhanced in the early phase (Fig. 1a), and a marginal contrast effect was observed in the late phase (Fig. 1b). The tumor was suspected to have originated from the sigmoid colon mesentery because the second sigmoidal artery (S2A) was detected as the feeding artery (Fig. 1c). There were no suspicious findings of invasion into neighboring organs, distant metastases, or lymph node metastases.

**Fig. 2:**
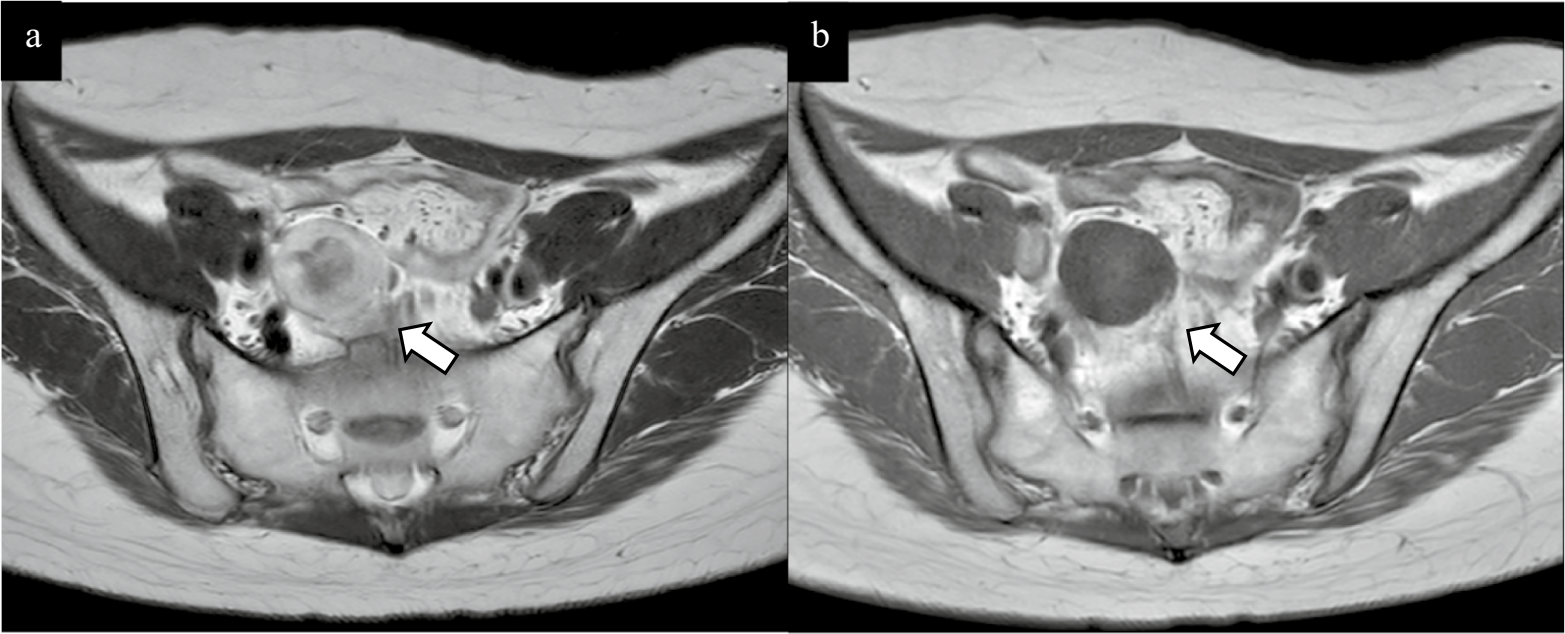
Abdominoopelvic magnetic resonance imaging (MRI). **a** Axial T2-weighted fast spin-echo MRI showed high signal at the limbus and mildly low signal in the interior. **b** Axial T1-weighted images showed heterogeneous low signal

**Fig. 3:**
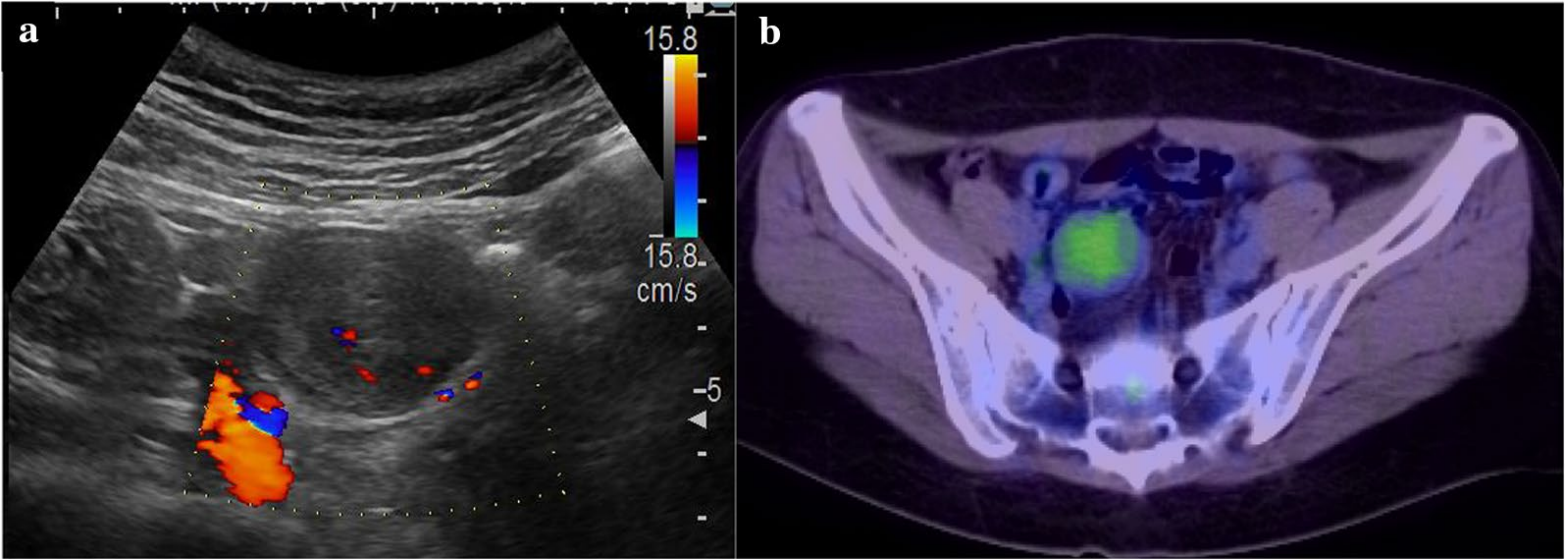
**a** Abdominal ultrasound image showing heterogeneous hypoechoic mass with a capsule and pulsatile internal blood flow. **b** Axial FDG PET–CT showing nodular accumulation in the pelvic region, but no distant metastases. *FDG PET–CT* fluorodeoxyglucose positron emission tomography–computed tomography

T2-weighted MRI images showed a high signal in the tumor margins and a low signal in the center (Fig. 2a). T1-weighted images showed a heterogeneous low signal. Abdominal ultrasonography revealed a hypoechoic mass with a clear margin, internal heterogeneity, and pulsatile internal blood flow, indicating a tumor with a capsule (Fig. 3a). Fluorodeoxyglucose (FDG) positron emission tomography–CT (PET–CT) showed nodular accumulation in the pelvis, with a maximum standardized uptake value of 3.2, and no distant metastases in the lymph nodes, lungs, or liver (Fig. 3b). Colonoscopy revealed no abnormalities.

### Preoperative diagnosis

The tumor in the present case was a well-defined, solid tumor with contrast enhancement. The differential diagnoses of the sigmoid mesenteric tumor in the present case included schwannoma, gastrointestinal stromal tumor (GIST), leiomyoma or leiomyosarcoma, desmoid tumor, and solitary fibrous tumor. Although leiomyoma was a possible differential diagnosis based on the patient’s history, we considered schwannoma to be the leading diagnosis based on the T2-weighted MRI because of the high signal intensity at the limbus and the mild low signal intensity internally.

As it was difficult to differentiate between benign and malignant tumors, we decided to resect the tumor en bloc without damaging the tumor capsule.

### Operative procedure and postoperative short-term outcomes

**Fig. 4:**
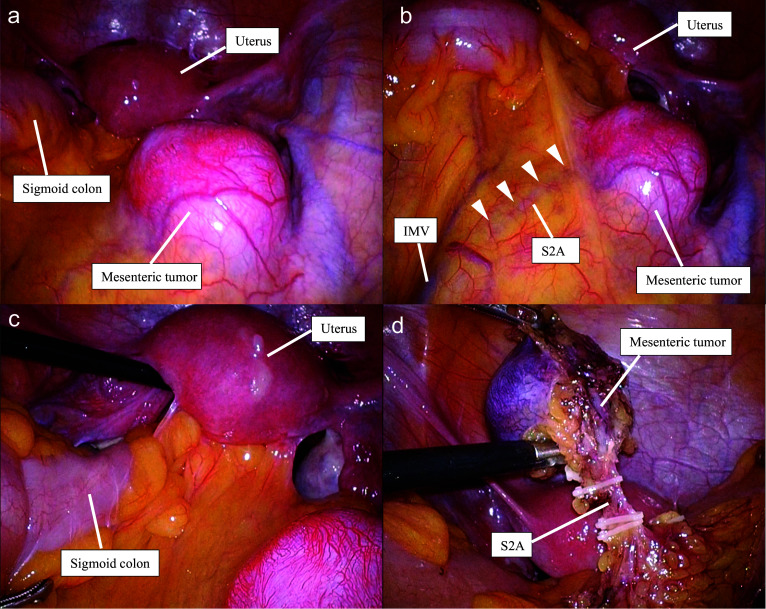
Surgical findings. **a** A relatively elastic soft tumor with erythematous tone was observed in the sigmoid mesentery. **b** Intraoperative findings suggested that the feeding artery was a branch of the S2A (arrow head), as diagnosed preoperatively. **c** Adhesions of the posterior uterus to the sigmoid colon was observed. **d** A branch of the S2A was clipped and the mesenteric tumor was resected en bloc. *S2A* second sigmoidal artery, *IMV* inferior mesenteric vein

We performed laparoscopic resection of a sigmoid colon mesenteric tumor. Twelve mm ports were inserted through the umbilicus and left lower abdomen, and 5 mm ports were inserted in the right lower abdomen and lateral abdomen. An erythematous, relatively elastic, and soft tumor was observed in the sigmoid mesentery; no other tumors were found (Fig. 4a). Based on the intraoperative findings, it was determined that the feeding artery was a branch of the S2A, as diagnosed preoperatively (Fig. 4b). During the procedure, adhesion of the posterior uterus to the sigmoid colon was observed, necessitating dissection of the adhesion (Fig. 4c). After securing the margin of the tumor and creating a half-surrounding incision through the serosa of the sigmoid mesentery, we confirmed that the tumor could be removed en bloc while preserving the blood flow in the sigmoid colon. After transecting the feeding artery, the tumor was successfully removed en bloc without compromising the tumor capsule (Fig. 4d). The operation time was 4 h and 15 min, and blood loss was minimal. The patient did not experience any postoperative complications and was discharged on postoperative day 7. There was no evidence of tumor recurrence on MRI 6 months after surgery.

### Histopathological findings

Macroscopically, the tumor measured 25 mm × 23 mm, and the cut surface was well demarcated with no hemorrhage or necrosis.

**Fig. 5:**
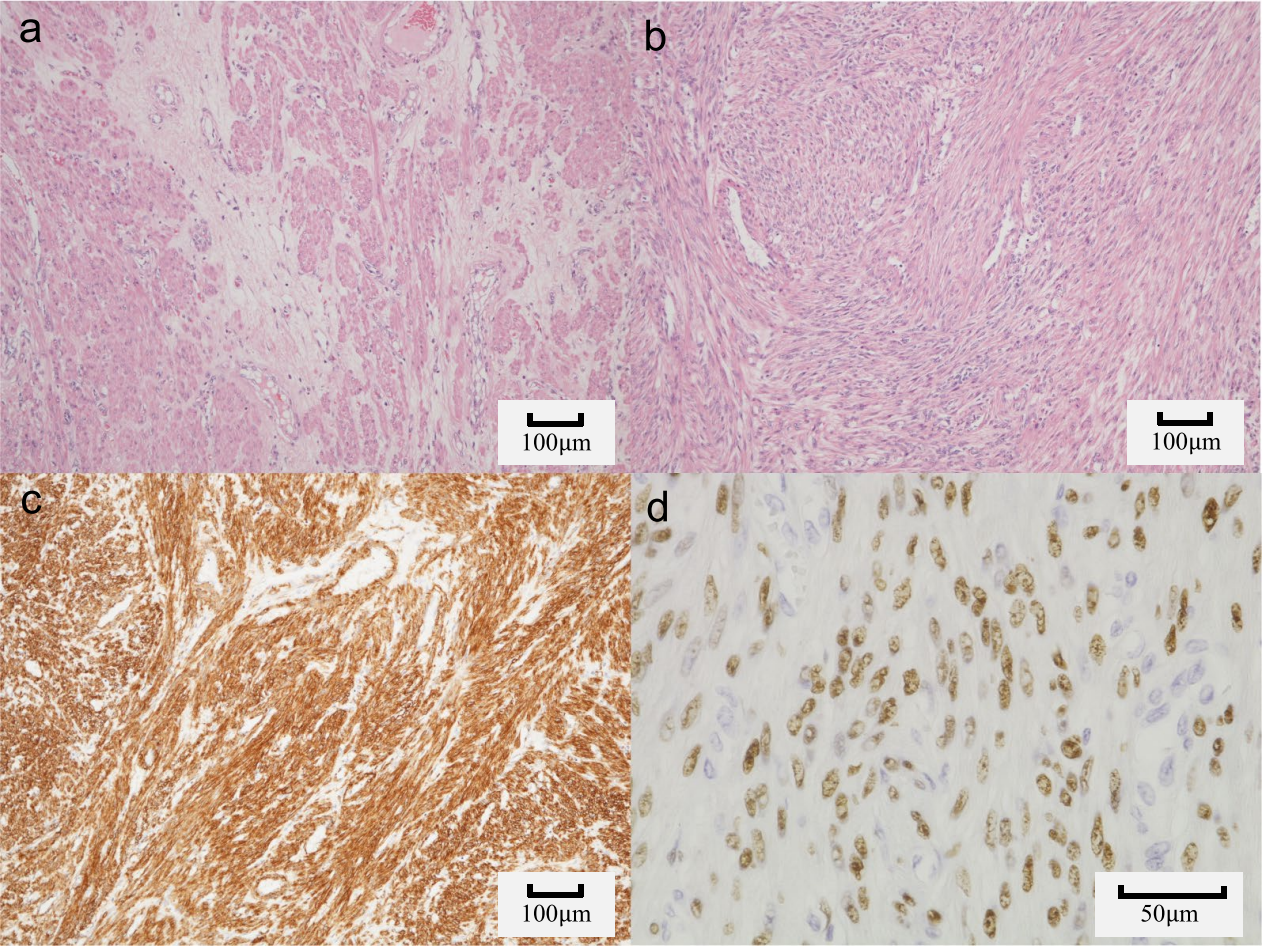
Histopathological finding. **a** Hematoxylin and eosin (HE) stain, ×10 (peripheral part); the cellular components are low and the interstitial component is prominent. **b** HE stain, ×10 (central part); rich cellular components and prominent vascular structure. **c** Alpha smooth muscle actin (α-SMA) positive, ×10. **d** Progesterone receptor (PgR) ×40, high PgR positivity. *HE* hematoxylin and eosin, *SMA* α-smooth muscle actin, *PgR* progesterone receptor

Hematoxylin and eosin staining revealed that the tumor was composed of spindle-shaped cells. The central part was rich in cellular components and contained many blood vessels (Fig. 5a). The marginal area showed edematous degeneration with a few tumor cells and sparse stromal components (Fig. 5b). No hemorrhage or necrosis was observed. The mitotic index was less than 1/50 (one per 50) high power field, and no malignant findings were observed.

Immunostaining findings of alpha-smooth muscle actin (α-SMA) (+) (Fig. 5c), desmin (+), c-kit (−), cluster of differentiation CD34 (−), discovered on GIST protein 1 (DOG1) (−), S-100 proteins (−), and CD68 (−) suggested leiomyoma of the sigmoid mesentery, and weak positivity for the estrogen receptor (ER) and strong positivity for the progesterone receptor (PgR) confirmed the pathological diagnosis of PL (Fig. 5d). Finally, the patient was diagnosed with PL of the sigmoid mesentery.

## Discussion

There is a wide variety of differential diagnoses for mesenteric tumors [4]. Imaging findings play an important role in the differential diagnosis of mesenteric tumors. A well-defined and solid tumor on CT and MRI, as in the present case, suggests mesenchymal tumors, such as schwannoma, GIST, leiomyoma or leiomyosarcoma, desmoid fibromatosis, and a solitary fibrous tumor. The present case also showed that the tumor had a high signal at the tumor margins on T2-weighted MRI images, which has been reported to be one of the specific findings of a schwannoma (target sign) [5]. Meanwhile, PL, which was the definite diagnosis in the present case, typically shows clear borderline findings with low signals on T2-weighted MRI images, similar to uterine myomas. This case demonstrates that PL can present with atypical imaging findings similar to those of abdominal mesenteric schwannomas.

Uterine myomas are histologically characterized by the growth of spindle-shaped cells composed of smooth muscle tissue in an intricate bundle-like pattern [6]. In contrast, uterine myomas are known to show various patterns of degeneration, such as hyaline, mucinous, calcified, cystic, fatty, and necrotic degeneration, and imaging findings can vary depending on the degeneration [7]. Similar to uterine myomas, it is conceivable that imaging findings would be atypical when tissue degeneration occurs [8]. In the present case, the high limbal signal observed on T2-weighted MRI was considered to reflect edematous degeneration, as the histopathological findings showed edematous degeneration with decreased cell density at the tumor margin. Schwannomas also have similar histological findings in that they have abundant cellular components in the center, which reflect low signals on T2-weighted images, and sparse cellular components, which reflect high signals on T2-weighted images [5]. Considering the imaging and histological findings of the present case and schwannoma, it is plausible that PL with edematous degeneration can show atypical imaging findings that mimic schwannoma on MR, with some reports about schwannoma-like uterine leiomyoma, and most cases reported that it was difficult to diagnose correctly in a preoperative setting [9].

This case also illustrates that PL should be considered as a tumor of the mesentery after laparoscopic myomectomy, even if it does not show typical imaging findings, such as uterine fibroids and no use of a morcellator. Motorized morcellators are widely used for shredding uterine myomas [10]. It has been reported as a risk factor for PL, although the overall incidence after laparoscopic myomectomy surgery is reported to be as low as 0.20%–1.25% [3, 8]. PL occurs at various sites. However, the most common location of parasitic myomas was reported to be the pelvis, above the paravesical, pararectal, and rectovaginal spaces [11–13]. A retrospective study reported that the majority (93%) of cases of PL occur in the pelvis [12]. Other locations include the intestines, peritoneum, and omentum, anterior abdominal wall, and trocar site [14–16]. There is insufficient evidence for an established technique for zero risk of PL after laparoscopic myomectomy because it is difficult to completely collect myoma fragments without remnants. In the present case, the patient underwent myomectomy using surgical scissors in a collecting bag instead of a morcellator. However, PL occurred. Although countermeasures have been implemented to mitigate the risk of PL, the possibility of PL should be considered when an intraperitoneal tumor is detected.

Unlike the diagnosis of uterine myoma, the diagnosis of schwannoma-like PL is presumed to be more challenging because of the absence of a connection between the lesion and the uterus.

## Conclusions

We report a case of PL of the sigmoid mesentery with schwannoma-like findings on imaging that was treated by laparoscopic surgery. PL is sometimes difficult to distinguish from schwannomas because of the variety of imaging findings, such as uterine fibroids. PL should be considered a tumor of the mesentery after laparoscopic myomectomy, even if it does not show typical imaging findings, such as uterine fibroids and no use of a morcellator.

## Data Availability

The data supporting the conclusions of this article are included within the article.

## Abbreviations

α-SMA: Alpha smooth muscle actin
CA19-9: Carbohydrate antigen 19-9
CEA: Carcinoembryonic antigen
CT: Computed tomography
DOG1: Discovered on GIST protein 1
ER: Estrogen receptor
FDG: Fluorodeoxyglucose
GIST: Gastrointestinal stromal tumor
MRI: Magnetic resonance imaging
PET: Positron emission tomography
PgR: Progesterone receptor
PL: Parasitic leiomyomas
sIL-2R: Soluble interleukin-2 receptor
S2A: Second sigmoidal artery
